# Supplementary material for: Tuning oxidant and antioxidant activities of ceria by anchoring copper single-site for antibacterial application
Source: Nat Commun. 2024 Feb 3;15:1010. doi: 10.1038/s41467-024-45255-6 (PMC10837451; doi:10.1038/s41467-024-45255-6)
Supplement: Supplementary file 1 — Supplementary Information [file 41467_2024_45255_MOESM1_ESM.pdf]

Supplementary Information

Tuning oxidant and antioxidant activities of ceria by anchoring copper single-site for antibacterial application

Peng Jiang,[1†] Ludan Zhang,[2†] Xiaolong Liu,[3†] Chenliang Ye,[1] Peng Zhu,[1] Ting Tan,[3*] Dingsheng Wang,[1*] and Yuguang Wang[2*]

[1] Department of Chemistry, Tsinghua University, Beijing 100084, China

[2] Center of Digital Dentistry/Department of Prosthodontics, National Center of Stomatology, National Clinical Research Center for Oral Diseases, National Engineering Laboratory for Digital and Material Technology of Stomatology, Beijing Key Laboratory of Digital Stomatology, NHC Research Center of Engineering and Technology for Computerized Dentistry, Peking University School and Hospital of Stomatology, Beijing 100081, China

[3] Laboratory of Theoretical and Computational Nanoscience, CAS Center for Excellence in Nanoscience, National Center for Nanoscience and Technology, Chinese Academy of Sciences, Beijing 100190, China

[†] P. Jiang, [†]L. Zhang, and [†]X. Liu contributed equally to this work.

*Correspondence to: tant@nanoctr.cn; wangdingsheng@mail.tsinghua.edu.cn; wangyuguang@bjmu.edu.cn.

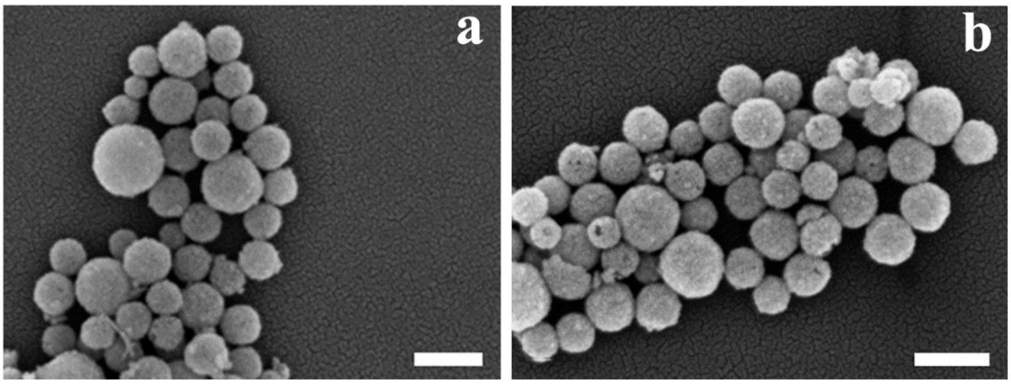

Supplementary Fig. 1. Morphology characterization of CeO_2 and Cu-CeO_2-air catalyst. Representative SEM images of (a) CeO_2 and (b) Cu-CeO_2-air catalyst (scale bar: 200 nm). A representative image of three replicates from each group is shown.

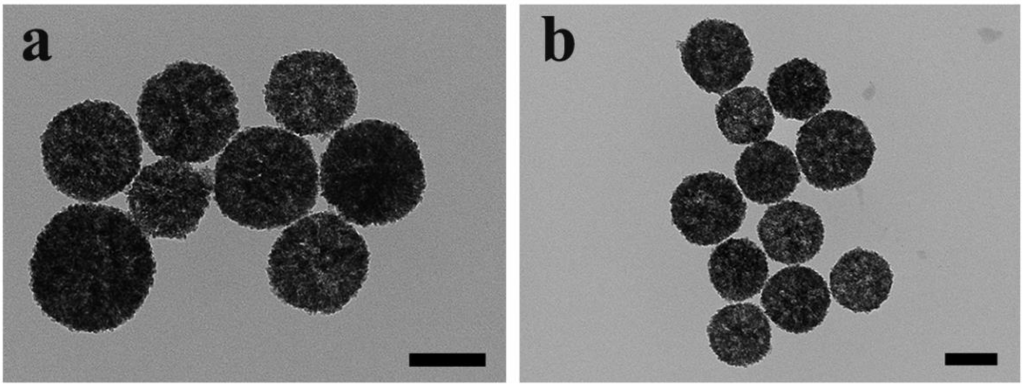

Supplementary Fig. 2. Morphology characterization of Cu-CeO_2 precursor and Cu-CeO_2-air catalyst. Representative TEM images of (a) Cu-CeO_2 precursor and (b) Cu-CeO_2-air catalyst (scale bar: 100 nm). The experiments were repeated three times with similar results.

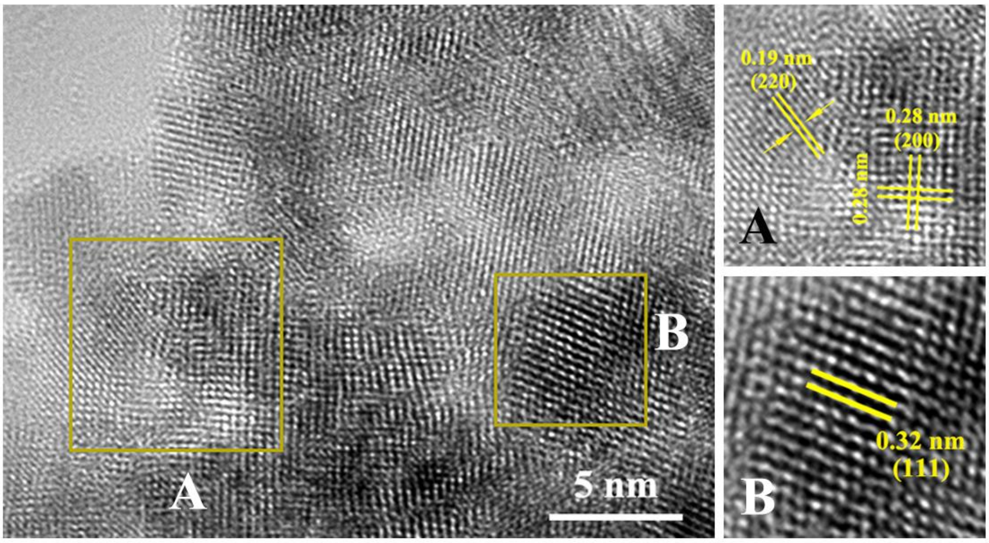

Supplementary Fig. 3. HRTEM images of Cu-CeO_2 precursor. A representative image of three replicates from each group is shown.

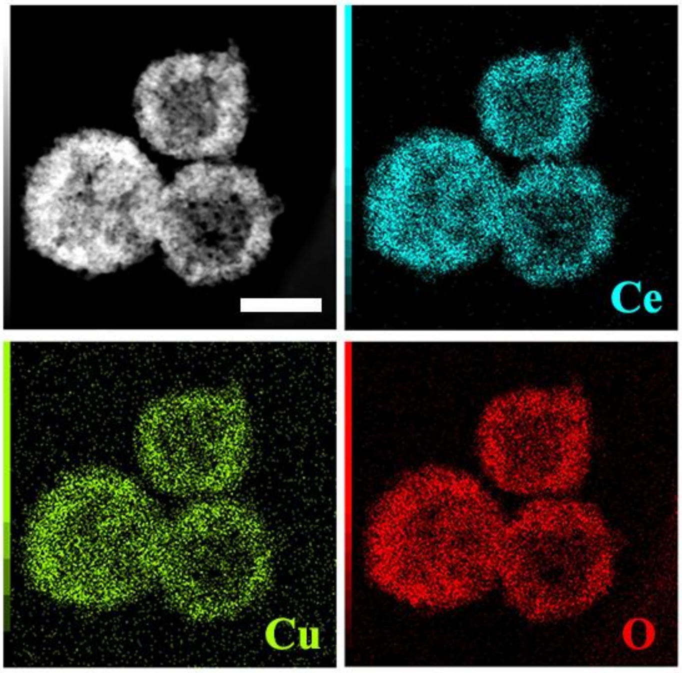

Supplementary Fig. 4. The elemental composition imaging analysis of Cu-CeO_2 precursor (scale bar: 50 nm). A representative image of three replicates from each group is shown.

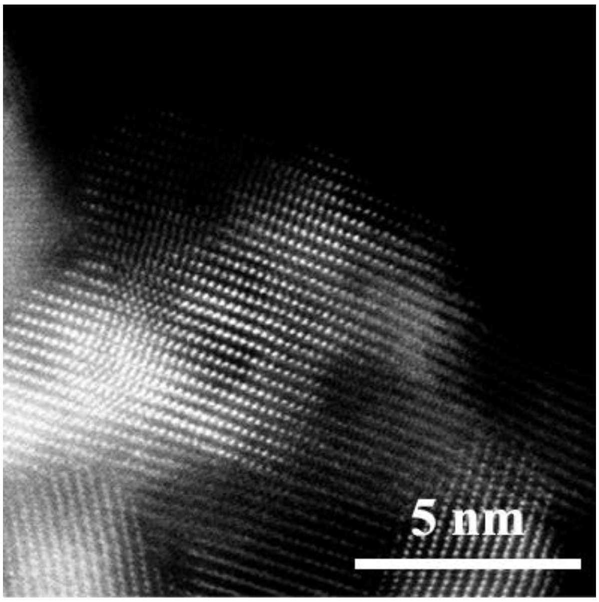

Supplementary Fig. 5. AC-HAADF-STEM image of Cu-CeO_2 precursor. A representative image of three replicates from each group is shown.

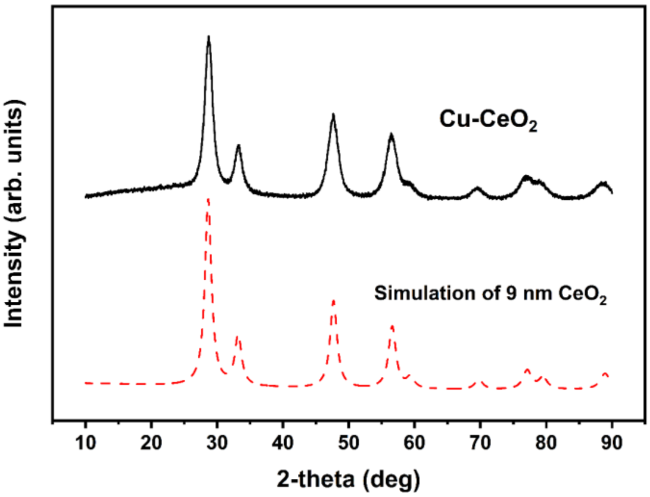

Supplementary Fig. 6. XRD fitting pattern of 9 nm Cu-CeO_2 sample. Source data are provided as a Source Data file.

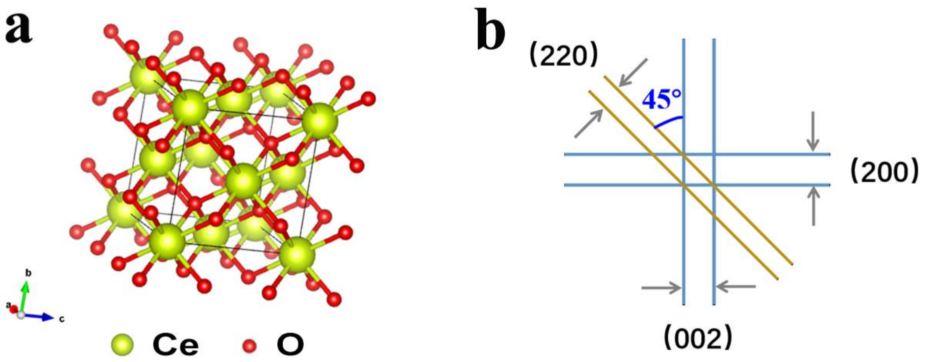

Supplementary Fig. 7. Structural illustration of cubic CeO_2. The model diagram of the crystal structure of cubic CeO_2 (a), the relationship between the spacing and the angle between the different crystal planes of the cubic lattice (b).

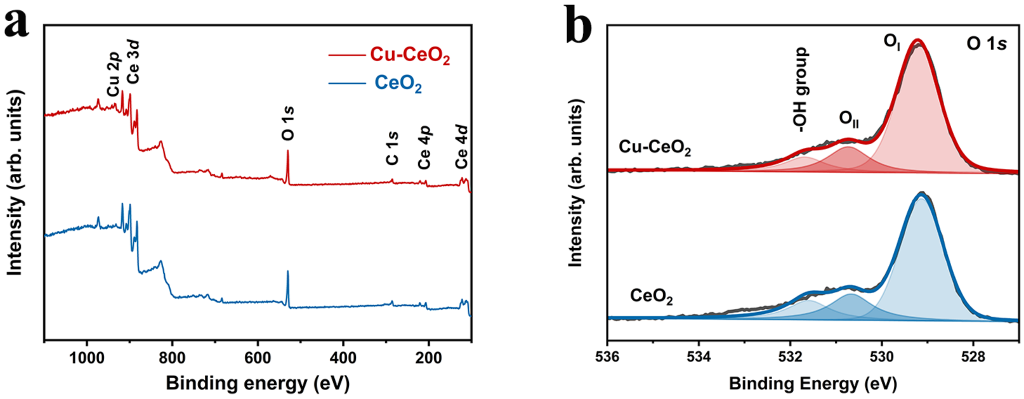

Supplementary Fig. 8. XPS spectra of Cu-CeO_2 and reference material. (a) XPS spectra of CeO_2 and Cu-CeO_2 samples, (b) O 1*s* photoelectron profiles of CeO_2 and Cu-CeO_2 catalysts. Source data are provided as a Source Data file.

Supplementary Table 1. The area ratios between Ce^{3+} and Ce^{4+} species of different samples in Figure 2c.

Sample	$Ce^{3+}/(Ce^{3+}+Ce^{4+})$
CeO_2	0.23
Cu-CeO_2	0.18

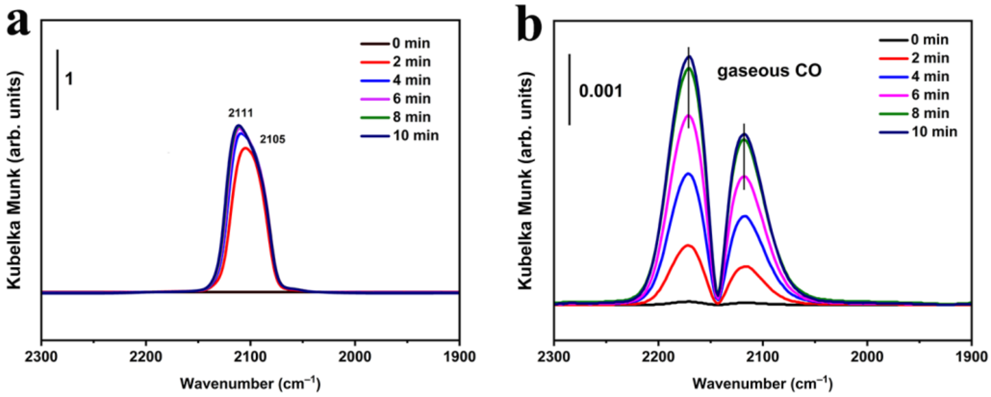

Supplementary Fig. 9. Study on Adsorption of CO on Cu-CeO_2 and CeO_2 samples. In situ DRIFTS study of CO adsorption on Cu-CeO_2 (a) and CeO_2 (b) samples. (2% CO/Ar flow rate, 30 mL min^{-1}; catalyst mass, 100 mg; temperature, 30 °C) Source data are provided as a Source Data file.

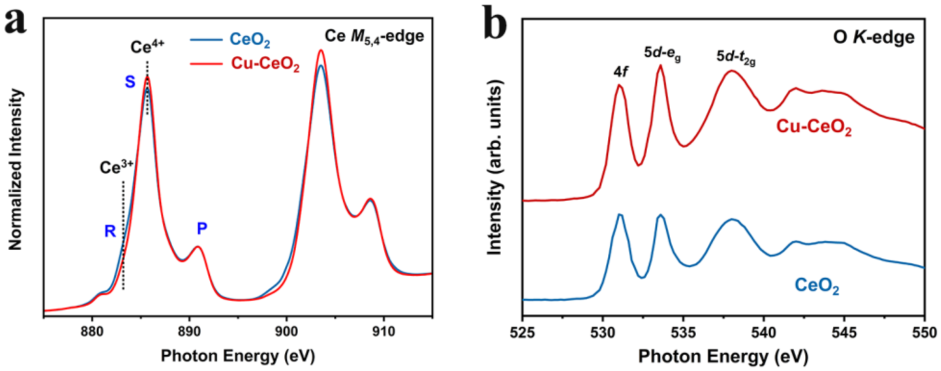

Supplementary Fig. 10. XANES spectra of CeO_2 and Cu-CeO_2 catalysts. (a) Soft XAS spectra of Ce $M_{4,5}$ absorption edges for CeO_2 and Cu-CeO_2 samples, (b) O K-edge XANES spectra for CeO_2 and Cu-CeO_2 catalysts. Source data are provided as a Source Data file.

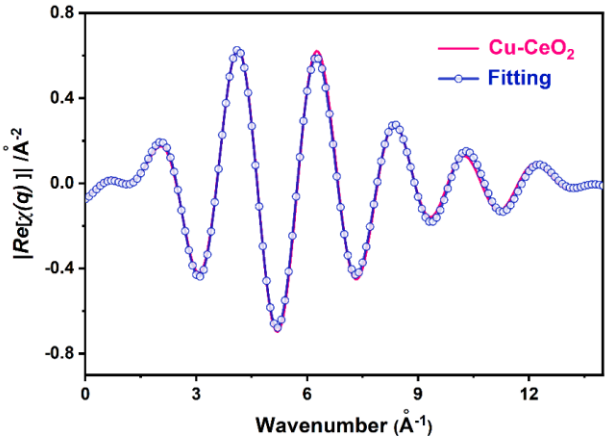

Supplementary Fig. 11. The q space fitting curve and experimental data of Cu-CeO_2. Source data are provided as a Source Data file.

Supplementary Table 2. Fitting results of FT-EXAFS spectra of $Cu\text{-}CeO_2$ sample at the Cu K-edge.

sample	Scattering path	Distance (Å)	C. N.	σ^2 ($Å^2$)	ΔE_0 (eV)	R-factor
**$Cu\text{-}CeO_2$**	Cu-O	1.94	3.2	0.004	-3.8	0.004

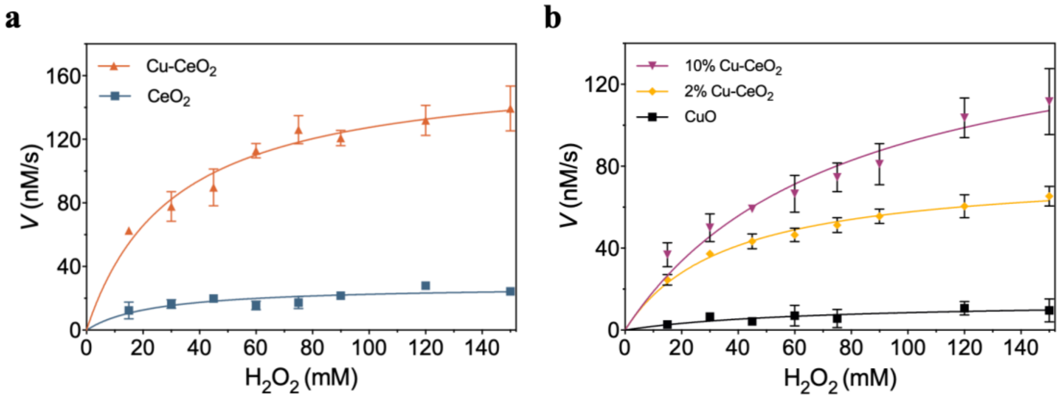

Supplementary Fig. 12. Steady-state kinetic assay of POD-like catalytic activity of CeO_2 and $Cu\text{-}CeO_2$ nanozymes with different Cu contents. Michaelis-Menten plot with different concentrations of H_2O_2 of (a) CeO_2, $Cu\text{-}CeO_2$ and (b) CuO, 2%, and 10% $Cu\text{-}CeO_2$. Data are presented as mean values +/- standard error of the mean, n = 3 independent replicates. Source data are provided as a Source Data file.

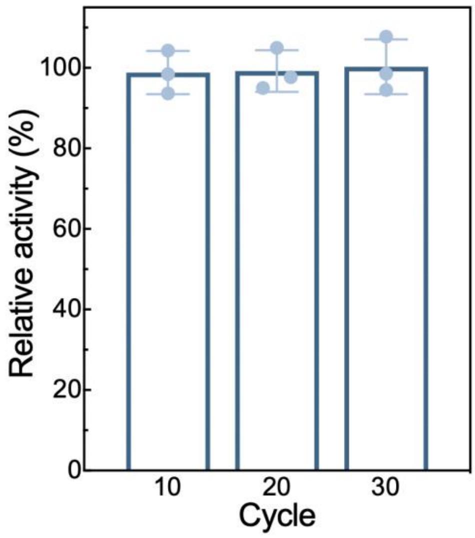

Supplementary Fig. 13. Relative cyclic stability of $Cu\text{-}CeO_2$. Data are presented as mean values +/- standard deviation, n = 3 independent replicates. Source data are provided as a Source Data file.

Supplementary Table 3. POD-like kinetic parameters of CeO_2 and Cu-CeO_2. (K_m: Michaelis-Menten constant, V_{max}: maximal reaction velocity)

Catalyst	V_{max} (nM/s)	K_m (mM)	*E* (M)	Turnover rate(/s)
CeO_2	28.05	24.34	-	-
CuO	14.15	65.86	6.24×10^{-4}	2.27×10^{-5}
2% Cu-CeO_2	78.34	35.94	1.47×10^{-5}	5.33×10^{-3}
5% Cu-CeO_2	166.7	30.76	3.46×10^{-5}	4.82×10^{-3}
10% Cu-CeO_2	161.4	75.83	7.03×10^{-5}	2.30×10^{-3}

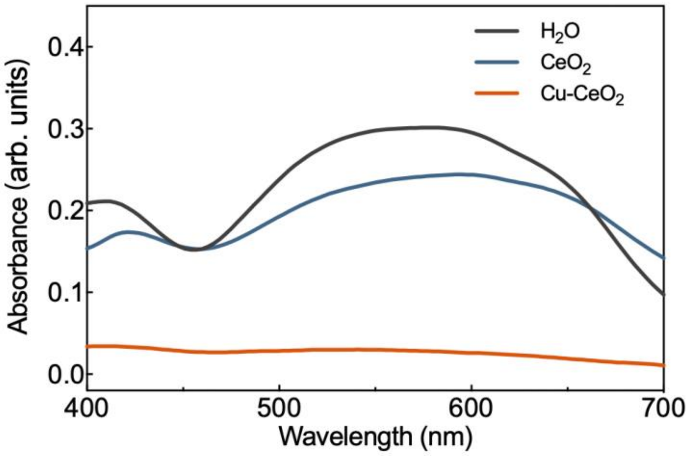

Supplementary Fig. 14. SOD-like catalytic activity of CeO_2 and Cu-CeO_2. Source data are provided as a Source Data file.

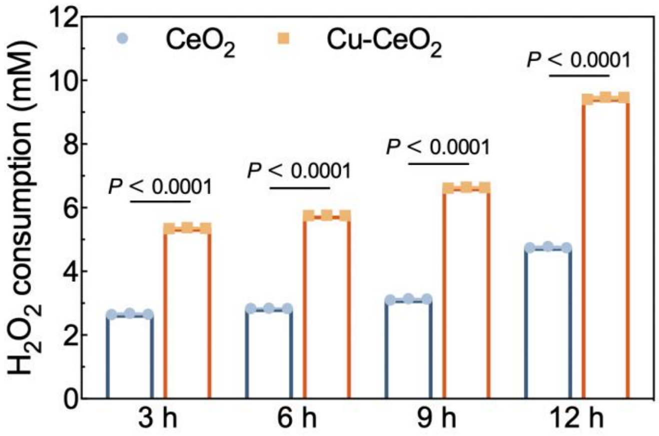

Supplementary Fig. 15. H_2O_2 consumption of CeO_2 and Cu-CeO_2. Data are presented as mean values +/- standard deviation, n = 3 independent replicates. Significance was calculated by two-sided Student's t-test. Source data are provided as a Source Data file.

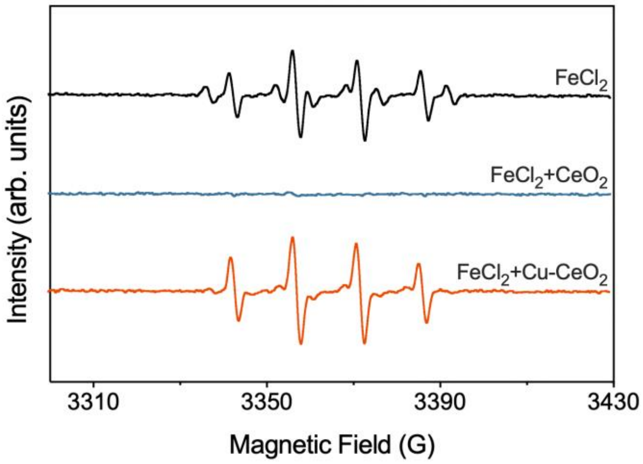

Supplementary Fig. 16. EPR spectrum of HORAC reactions of CeO_2 and Cu-CeO_2. Source data are provided as a Source Data file.

Supplementary Table 4. Quantitive countings of ERP spins of HORAC reactions of CeO_2 and Cu-CeO_2.

Group	Spins	Spin concentration (M)
$FeCl_2$	3.91×10^{13}	3.64×10^{-6}
$FeCl_2$+CeO_2	1.07×10^{12}	9.98×10^{-8}
$FeCl_2$+Cu-CeO_2	6.20×10^{13}	5.77×10^{-8}

Supplementary Table 5. DFT calculated reaction energy (in eV) of POD-like (P1-P4) and HORAC (H1-H6) processes on different reaction sites.

Reaction	Reaction Sites					
	Ce7c	Ce7c@ Cu-ad	Cu@ Cu-ad	Cu@ Cu-sub	diCe6c@ Cu-sub	Ce7c@ Cu-sub
P1	-0.952	-1.046	-0.986	-1.442	-1.123	-0.760
P2	2.591	1.792	1.358	2.479	0.835	1.556
P3	-1.064	-0.373	-0.010	-0.617	0.597	-0.082
P4	0.704	0.906	0.917	0.860	0.970	0.566
H1	-1.416	-2.309	-2.683	-2.019	-3.343	-2.234
H2	-3.292	-1.748	-2.574	-1.674	-2.265	-1.542
H3	0.909	1.258	1.110	0.652	0.746	0.924
H4	-4.711	-6.359	-4.946	-6.112	-4.888	-6.296
H5	0.299	0.914	0.737	0.606	0.700	0.663
H6	0.114	0.148	0.261	0.450	0.954	0.390

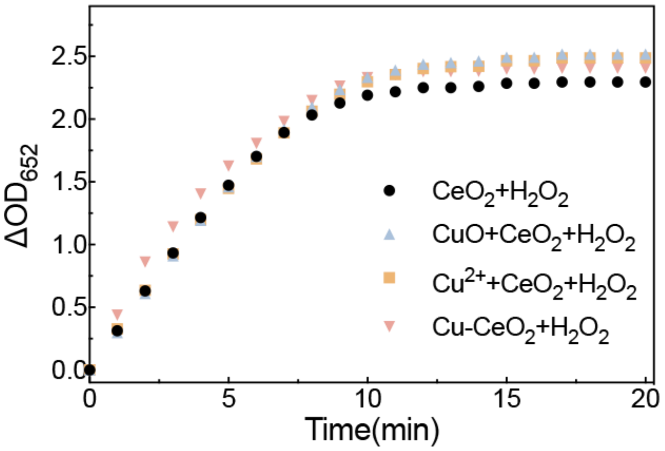

Supplementary Fig. 17. Time-dependent optical density change at 652 nm of TMB in POD reactions. Source data are provided as a Source Data file.

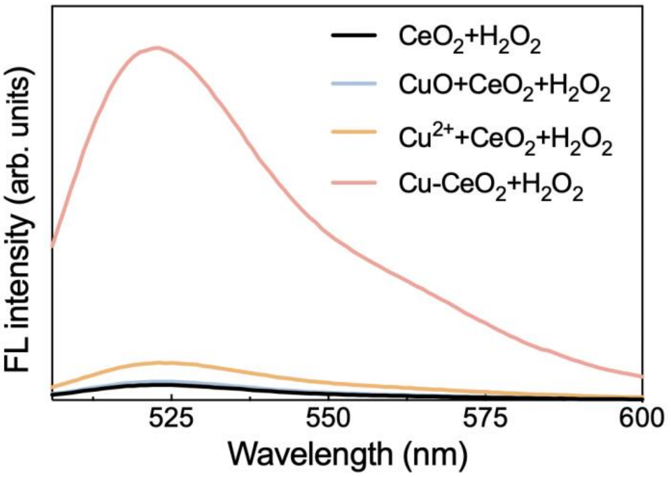

Supplementary Fig. 18. Fluorescent spectra of DCFH after 10 min reaction with H_2O_2 and different nanozymes. Source data are provided as a Source Data file.

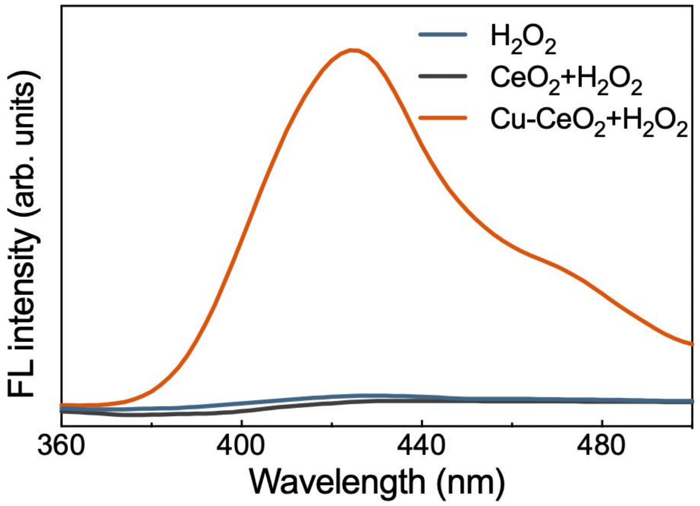

Supplementary Fig. 19. Fluorescent spectra of TA after 30 min reaction with H_2O_2 and different nanozymes. Source data are provided as a Source Data file.

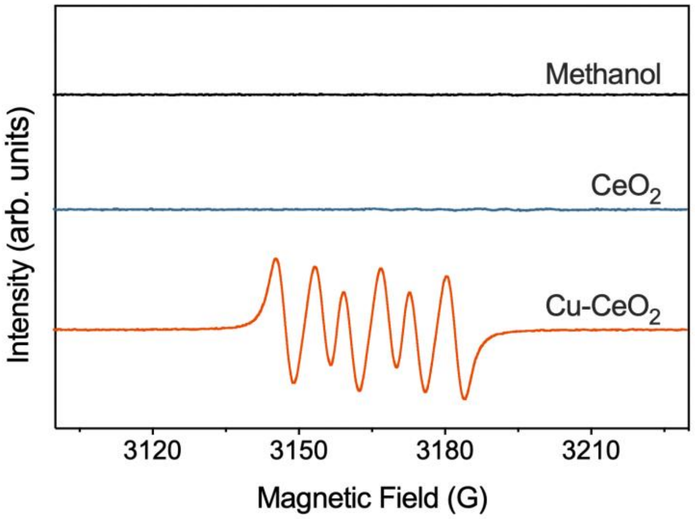

Supplementary Fig. 20. EPR spectrum of OXD reactions of CeO_2 and $Cu\text{-}CeO_2$. Source data are provided as a Source Data file.

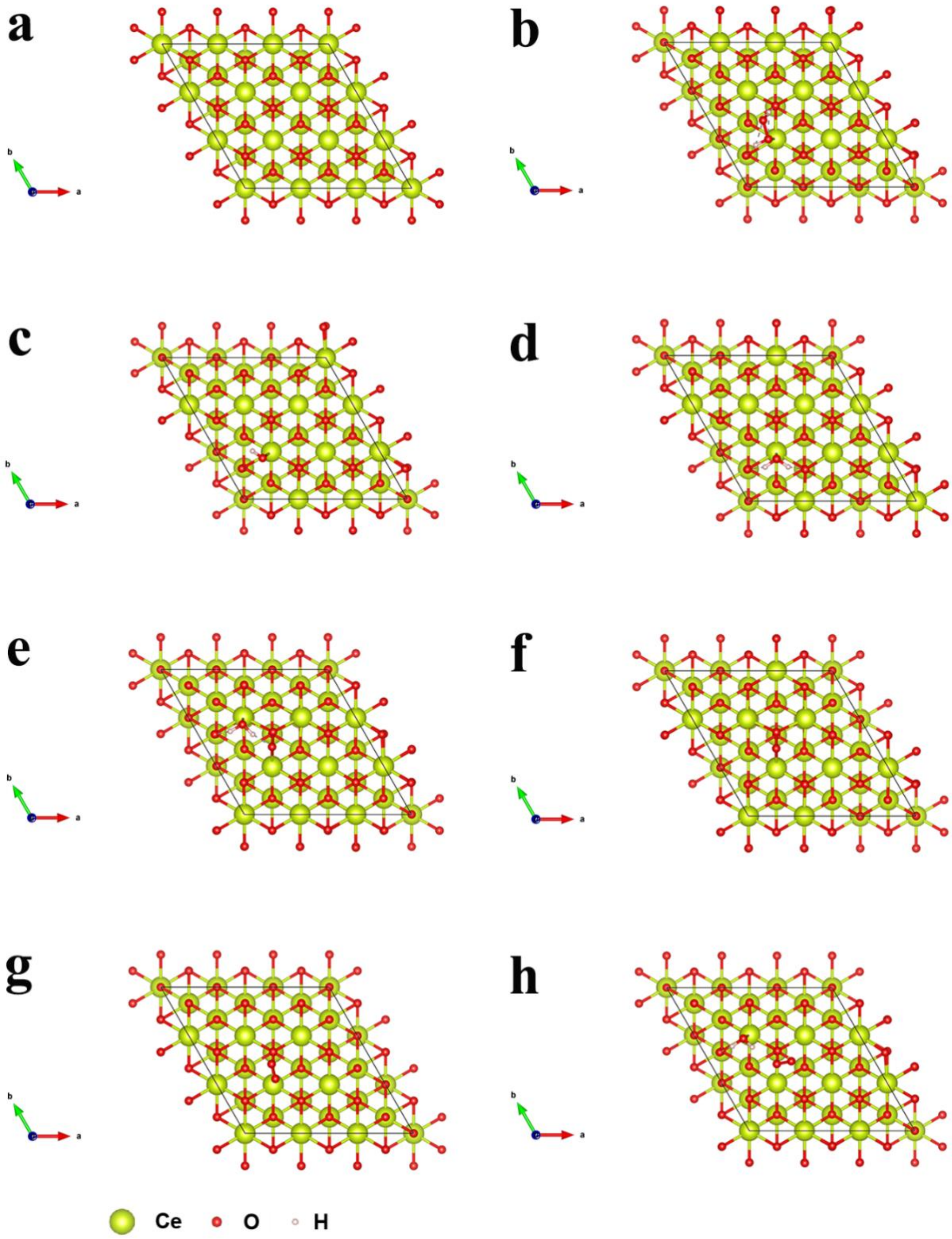

Supplementary Fig. 21. The DFT optimized geometry of reaction intermediates on Ce7c site. (a) clean slab, (b) $*H_2O_2$, (c) *OH, (d) $*H_2O$, (e) $*H_2O$ + *O, (f) *O, (g) $*O_2$, (h) $*H_2O$ + $*O_2$. Source data are provided as a Supplementary Data 1 file.

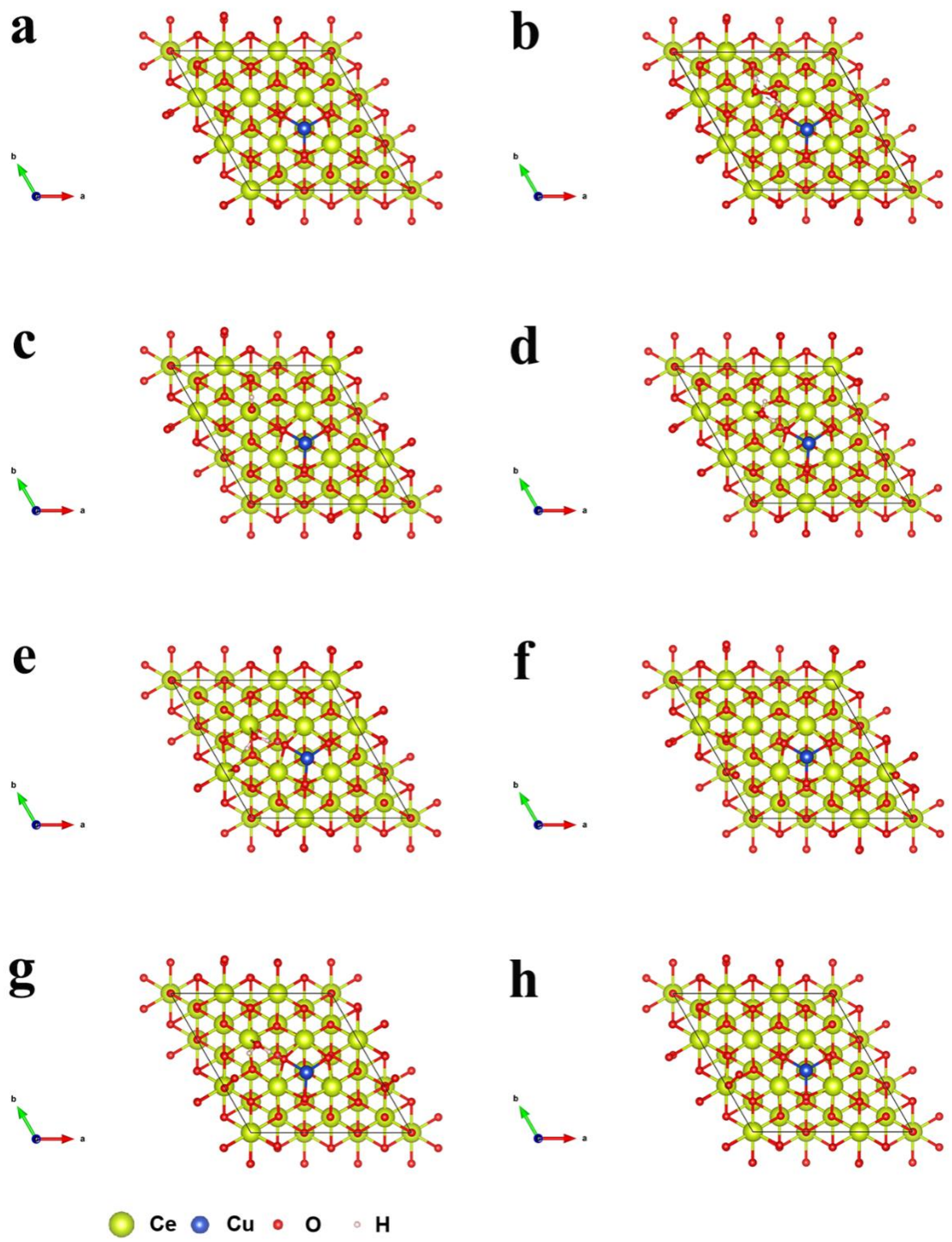

Supplementary Fig. 22. The DFT optimized geometry of reaction intermediates on Ce7c@Cu-ad site. (a) clean slab, (b) *H_2O_2, (c) *OH, (d) *H_2O, (e) *H_2O + *O, (f) *O, (g) *H_2O + *O_2, (h) *O_2. Source data are provided as a Supplementary Data 1 file.

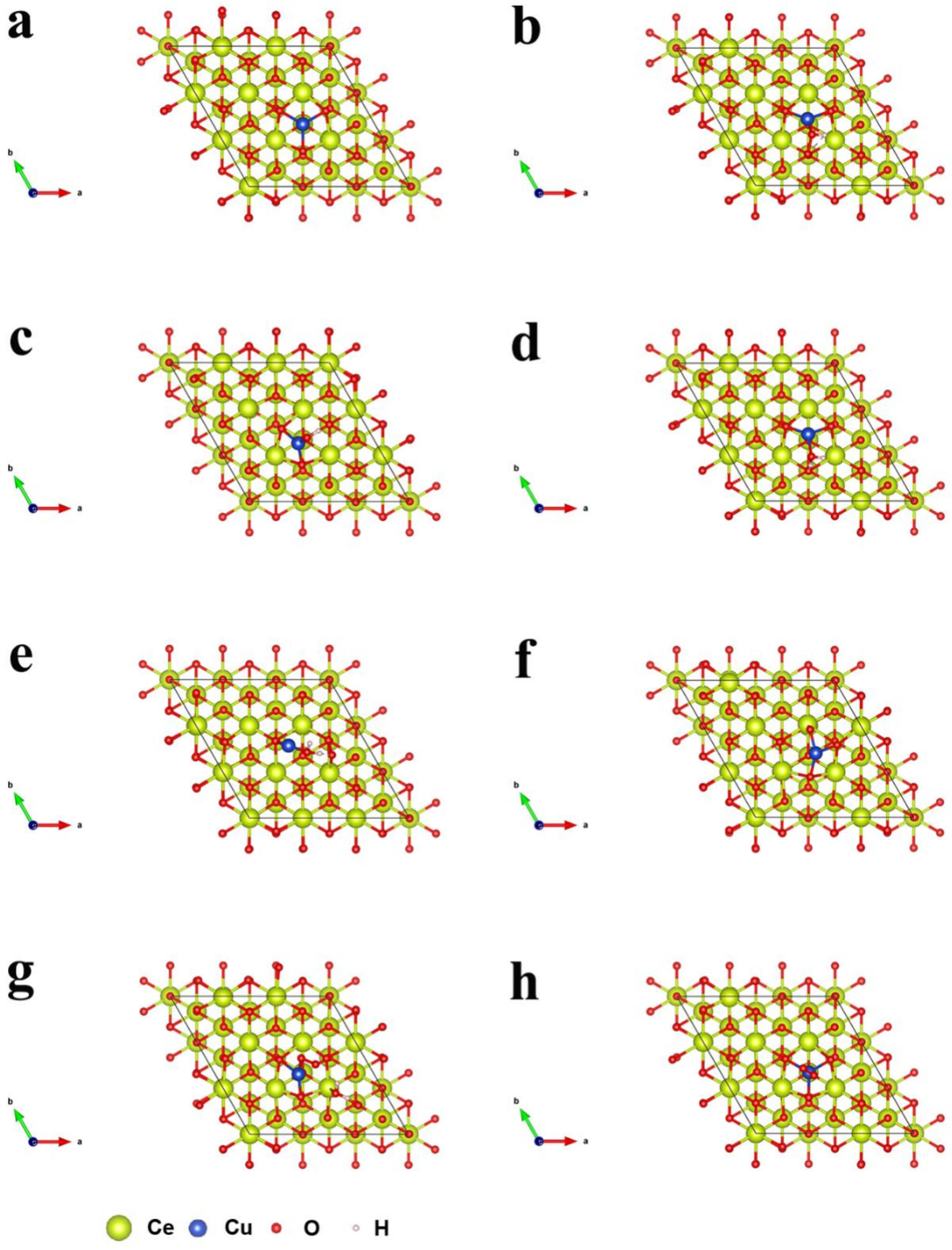

Supplementary Fig. 23. The DFT optimized geometry of reaction intermediates on Cu@Cu-ad. (a) clean slab, (b) *H_2O_2, (c) *OH, (d) *H_2O, (e) *H_2O + *O, (f) *O, (g) *H_2O + *O_2, (h) *O_2. Source data are provided as a Supplementary Data 1 file.

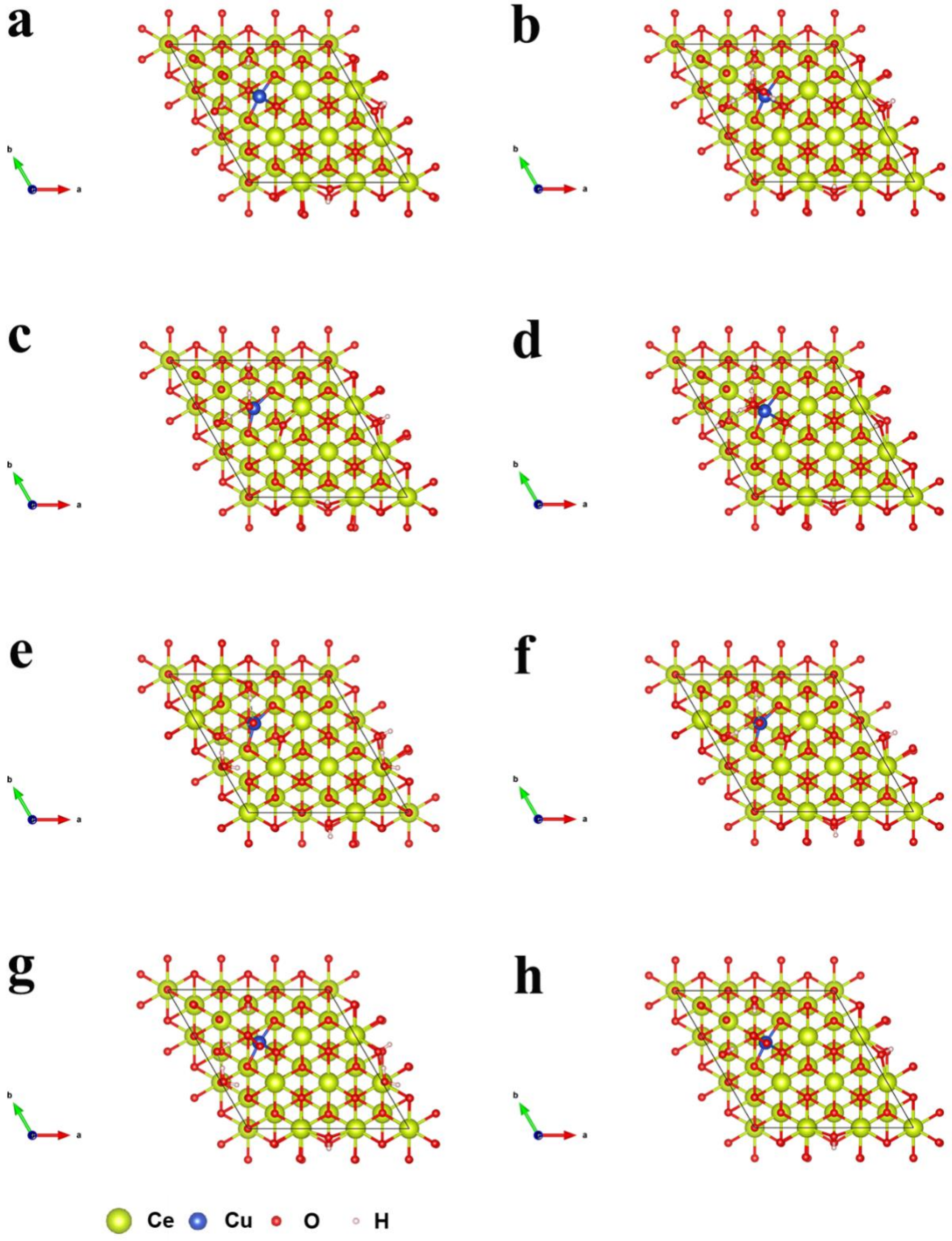

Supplementary Fig. 24. The DFT optimized geometry of reaction intermediates on Cu@Cu-sub. (a) clean slab, (b) *H_2O_2, (c) *OH, (d) *H_2O, (e) *H_2O + *O, (f) *O, (g) *H_2O + *O_2, (h) *O_2. Source data are provided as a Supplementary Data 1 file.

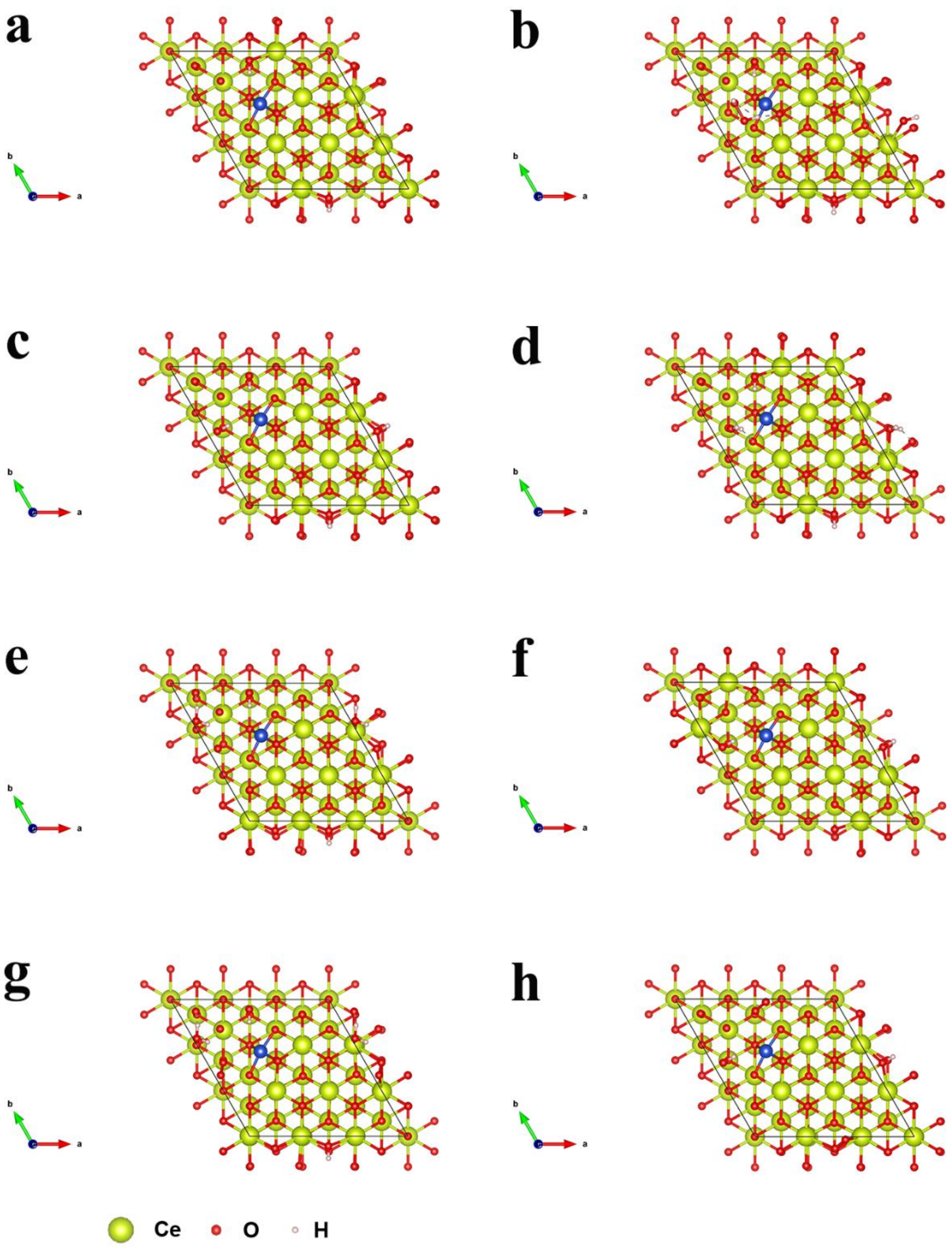

Supplementary Fig. 25. The DFT optimized geometry of reaction intermediates on diCe6c@Cu-sub. (a) clean slab, (b) *H_2O_2, (c) *OH, (d) *H_2O, (e) *H_2O + *O, (f) *O, (g) *H_2O + *O_2, (h) *O_2. Source data are provided as a Supplementary Data 1 file.

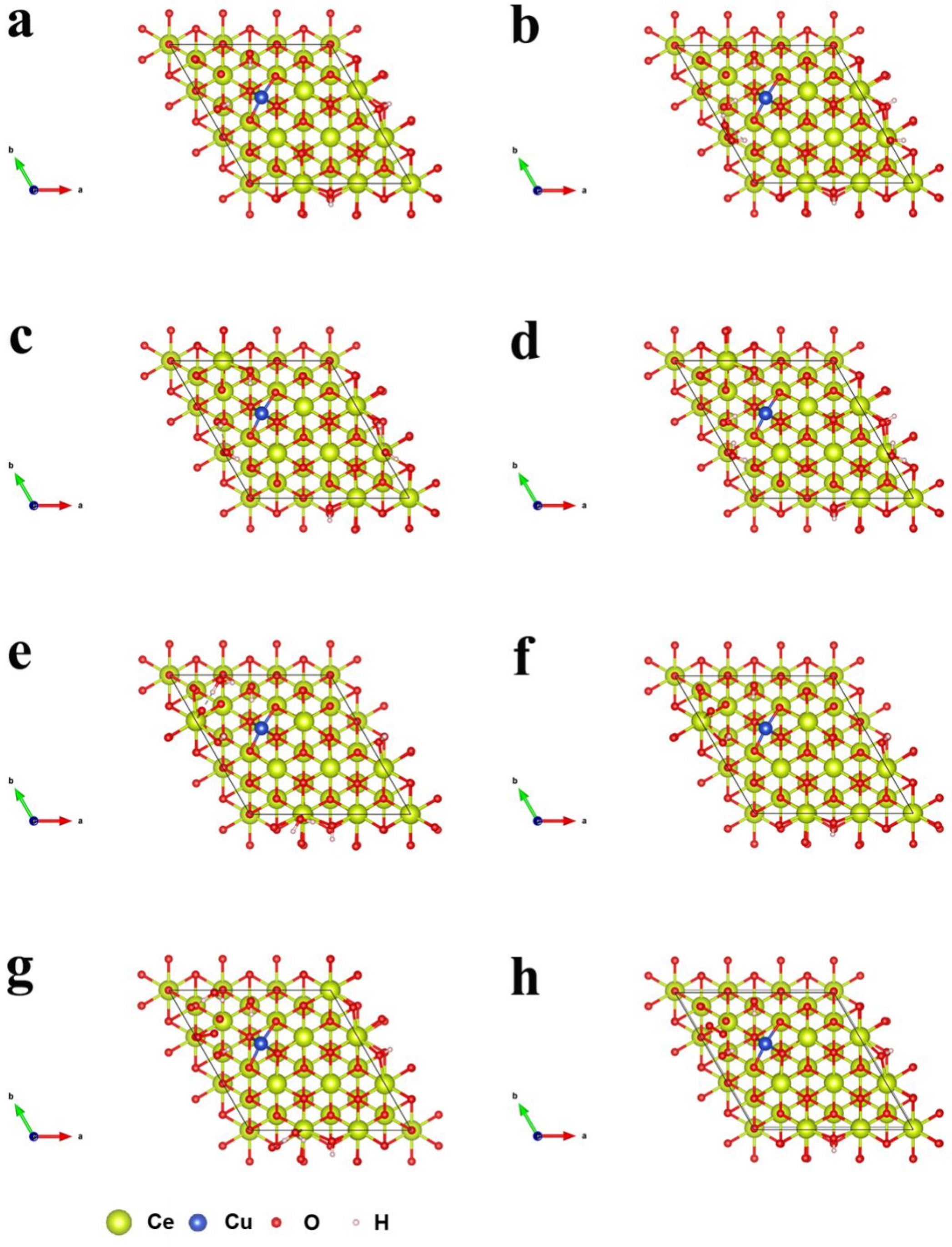

Supplementary Fig. 26. The DFT optimized geometry of reaction intermediates on Ce7c@Cu-sub. (a) clean slab, (b) *H_2O_2, (c) *OH, (d) *H_2O, (e) *H_2O + *O, (f) *O, (g) *H_2O + *O_2, (h) *O_2. Source data are provided as a Supplementary Data 1 file.

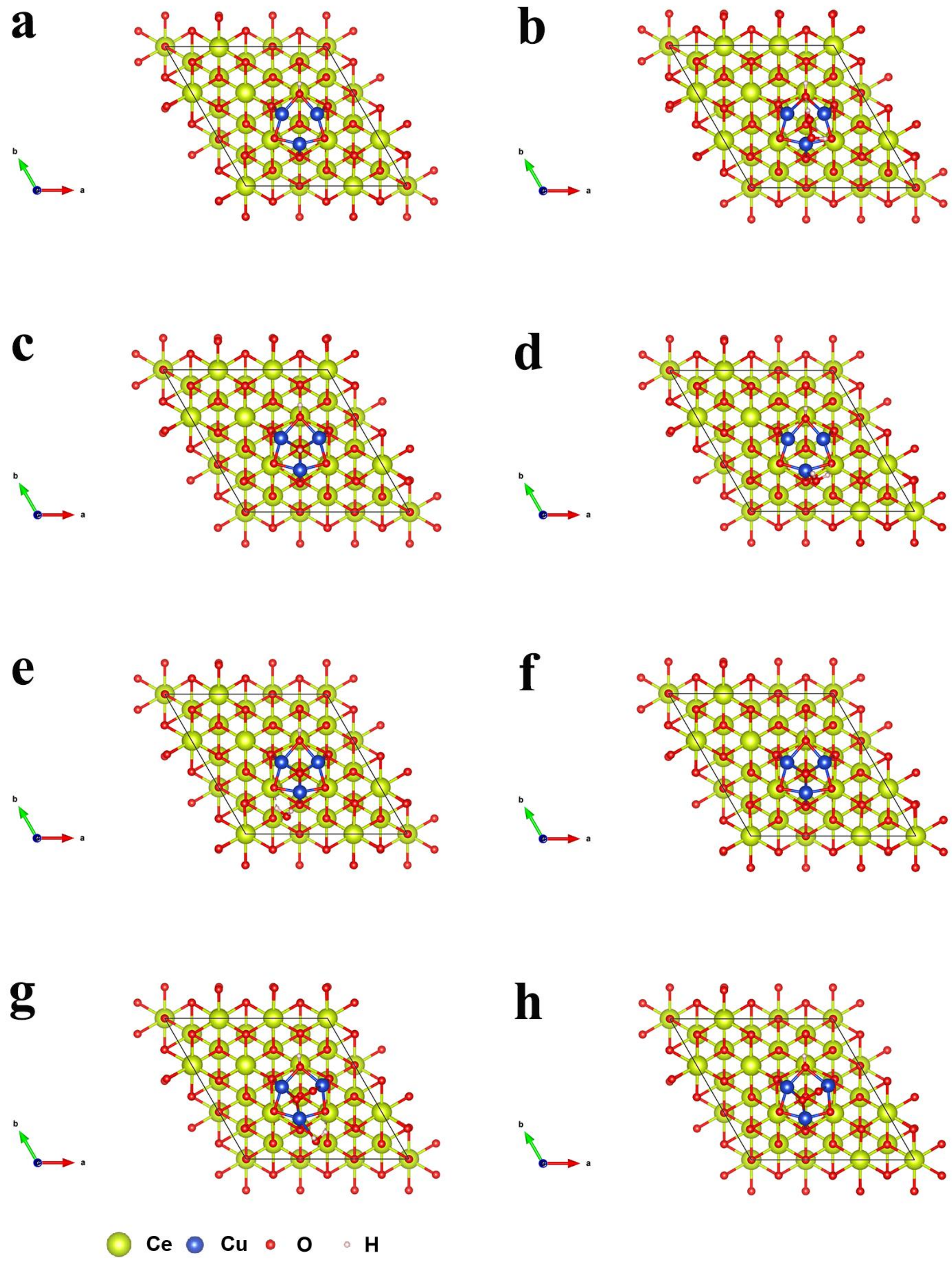

Supplementary Fig. 27. The DFT optimized geometry of reaction intermediates on Cu_3 site. (a) clean slab, (b) $*H_2O_2$, (c) *OH, (d) $*H_2O$, (e) $*H_2O$ + *O, (f) *O, (g) $*O_2$, (h) $*H_2O$ + $*O_2$. Source data are provided as a Supplementary Data 1 file.

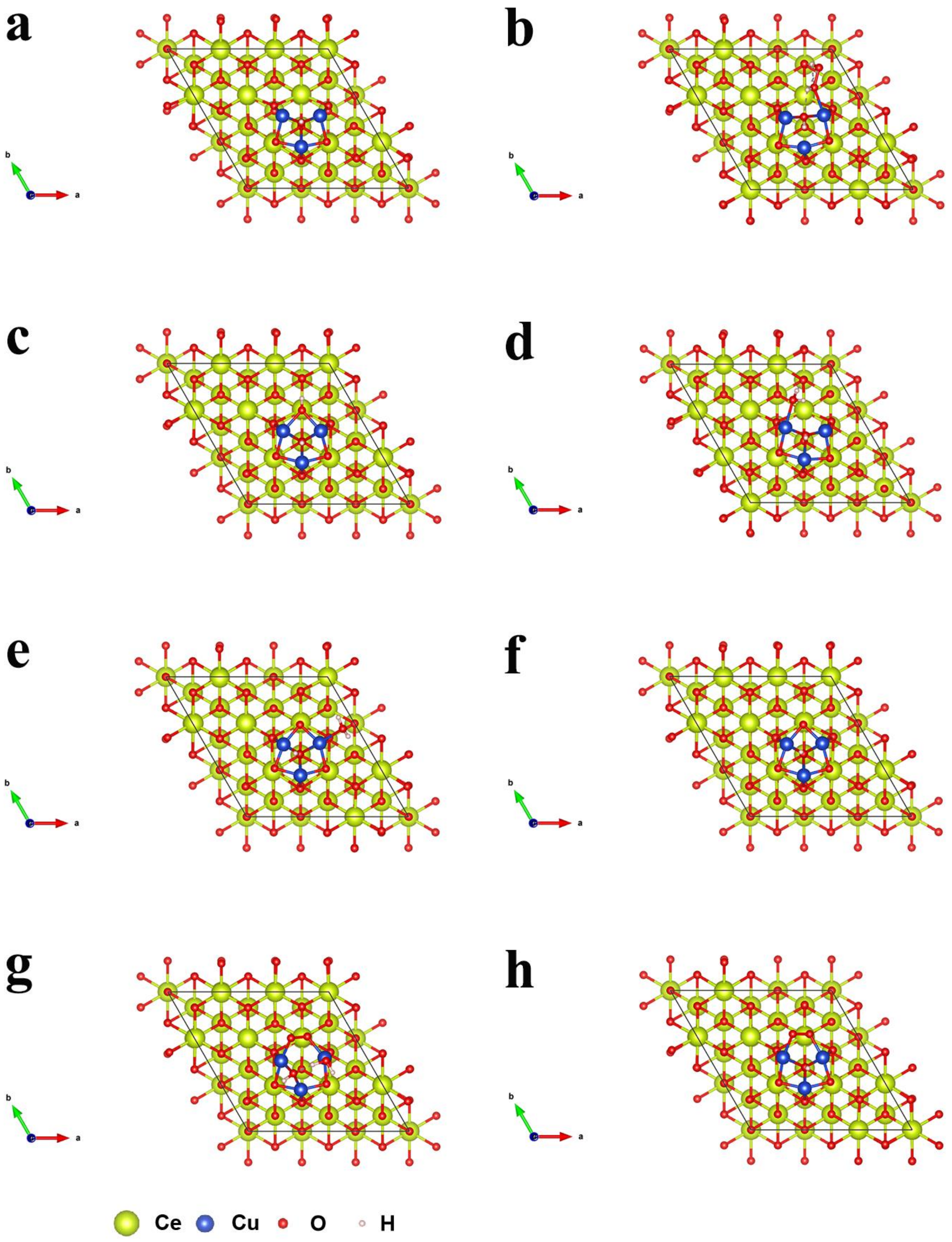

Supplementary Fig. 28. The DFT optimized geometry of reaction intermediates on Cu_2Ce site. (a) clean slab, (b) $*H_2O_2$, (c) *OH, (d) $*H_2O$, (e) $*H_2O$ + *O, (f) *O, (g) $*O_2$, (h) $*H_2O$ + $*O_2$. Source data are provided as a Supplementary Data 1 file.

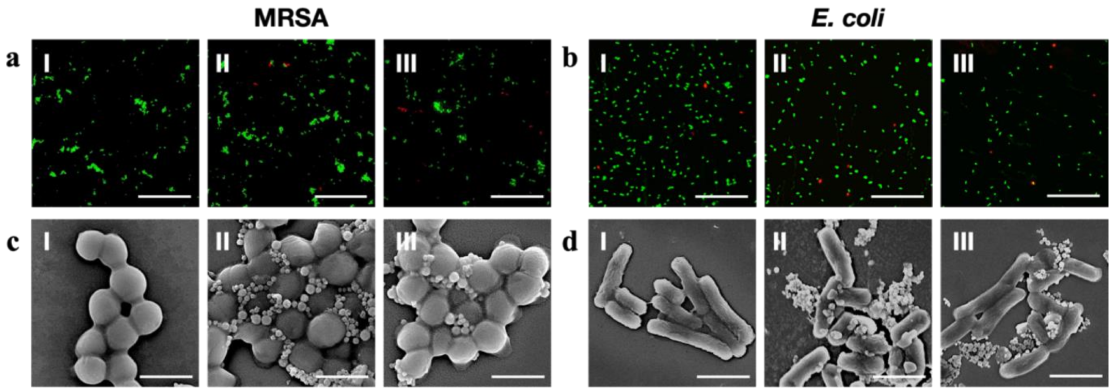

Supplementary Fig. 29. In vitro antibacterial performance of the nanozymes. Fluorescent images (scale bar: 50 μm) and SEM images (scale bar: 1 μm) of MRSA (a, c) and *E. coli* (b, d) after grouped treatment (I: PBS, II: CeO_2, III: Cu-CeO_2). A representative image of three replicates from each group is shown.

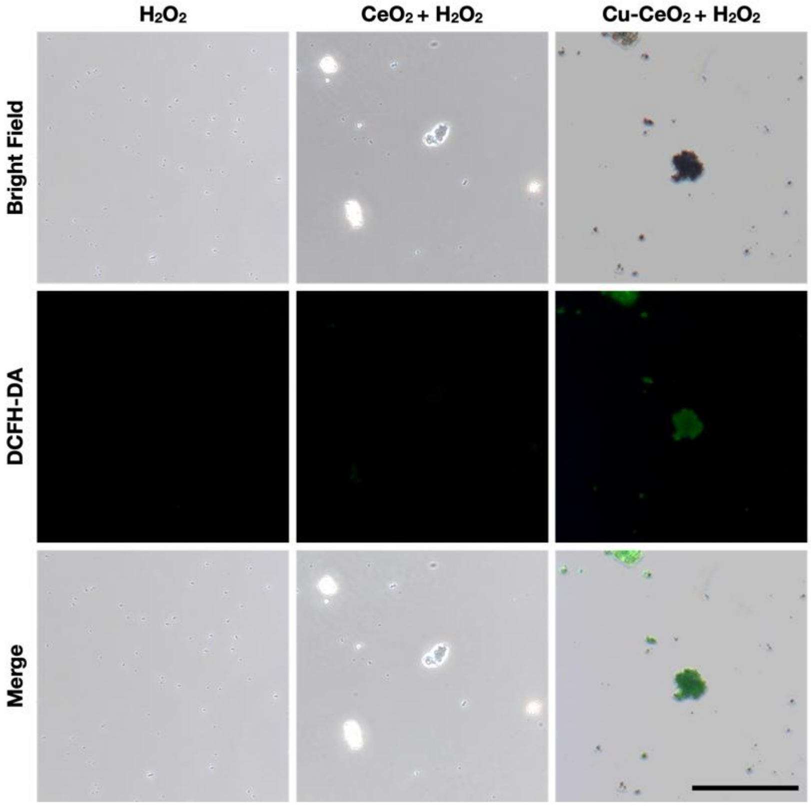

Supplementary Fig. 30. ROS fluorescent staining of MRSA after different treatments. Scale bar: 50 μm. A representative image of three replicates from each group is shown.

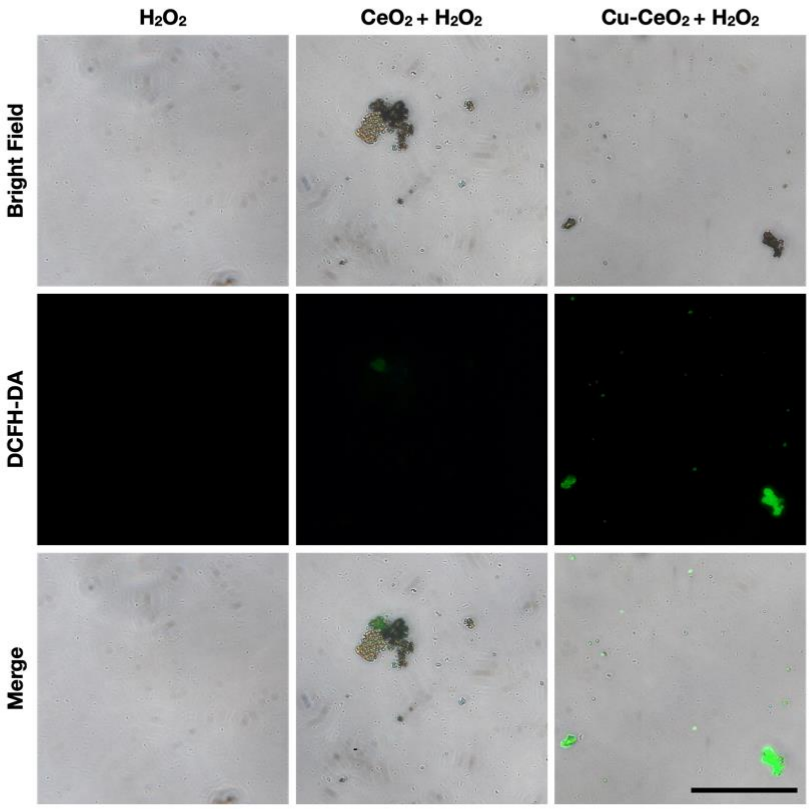

Supplementary Fig. 31. ROS fluorescent staining of *E. coli* after different treatments. Scale bar: 50 µm. A representative image of three replicates from each group is shown.

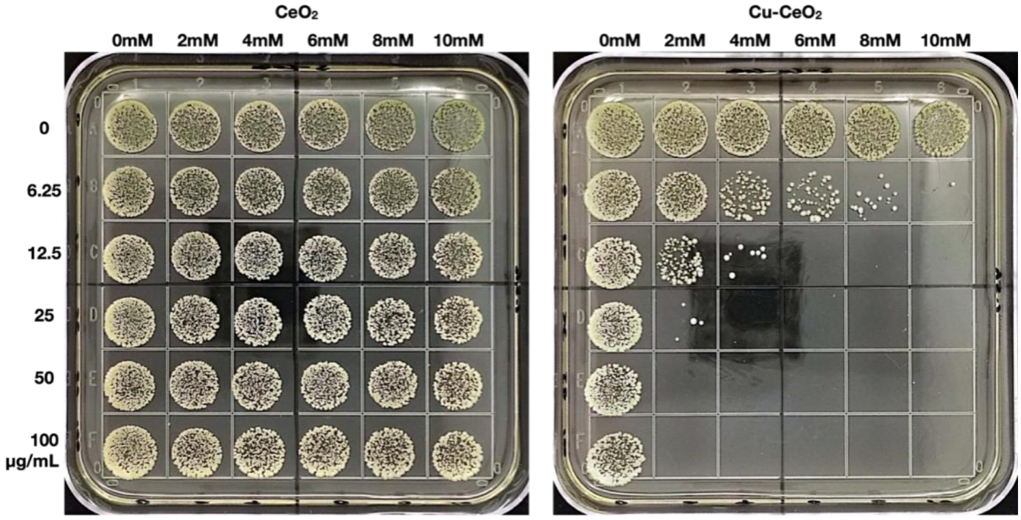

Supplementary Fig. 32. Bacterial colonies of MRSA after treatment with different concentrations of CeO_2, Cu-CeO_2, and H_2O_2. A representative image of three replicates from each group is shown.

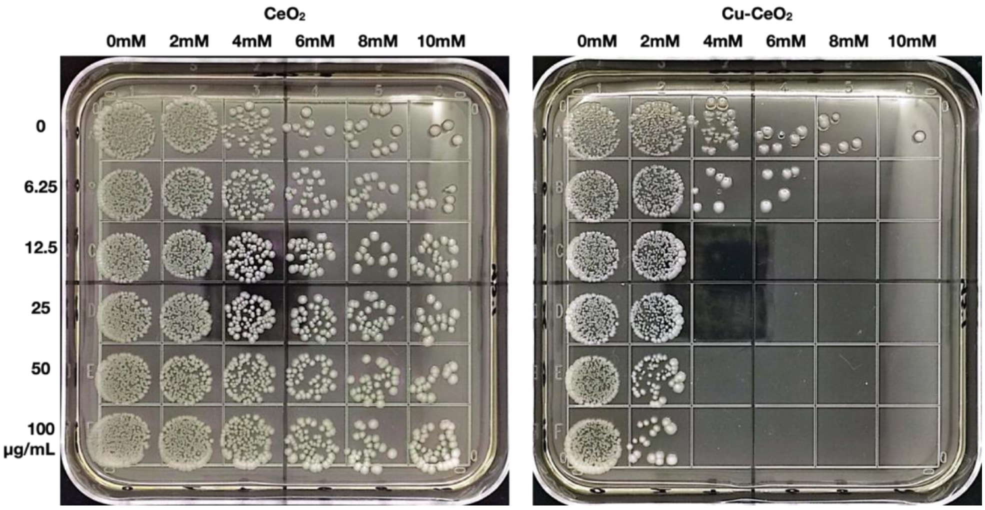

Supplementary Fig. 33. Bacterial colonies of *E. coli* after treatment with different concentrations of CeO_2, Cu-CeO_2 and H_2O_2. A representative image of three replicates from each group is shown.

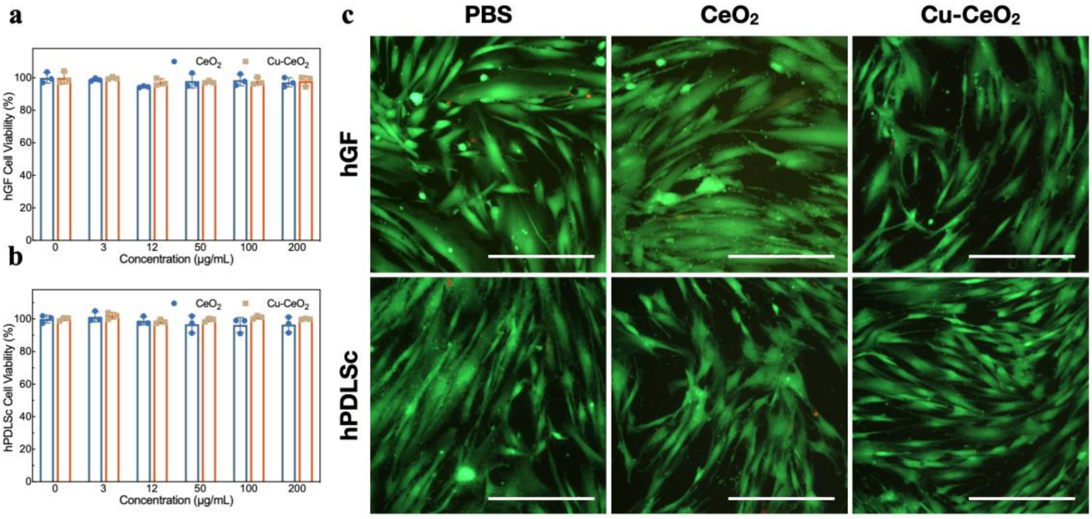

Supplementary Fig. 34. Biocompatibility of CeO_2 and Cu-CeO_2. Relative cell viability of hGF (a) and hPDLSc (b) after treatment with different concentrations of nanozymes. (c) Fluorescent images of hGF and hPDLSc after grouped treatment. Scale bar: 200 μm. A representative image of three replicates from each group is shown. Data are presented as mean values +/- standard deviation, n = 3 biologically independent replicates. Source data are provided as a Source Data file.

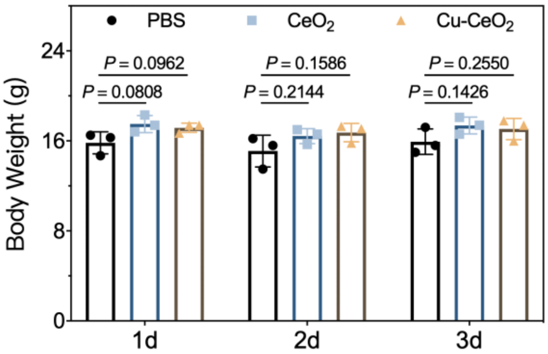

Supplementary Fig. 35. Body weights of balb/c mice after intravenous injection with PBS, CeO_2 and Cu-CeO_2 (n.s., $P > 0.05$, n=3). Data are presented as mean values +/- standard deviation, n = 3 biologically independent replicates. Significance was calculated by two-sided Student's t-test. Source data are provided as a Source Data file.

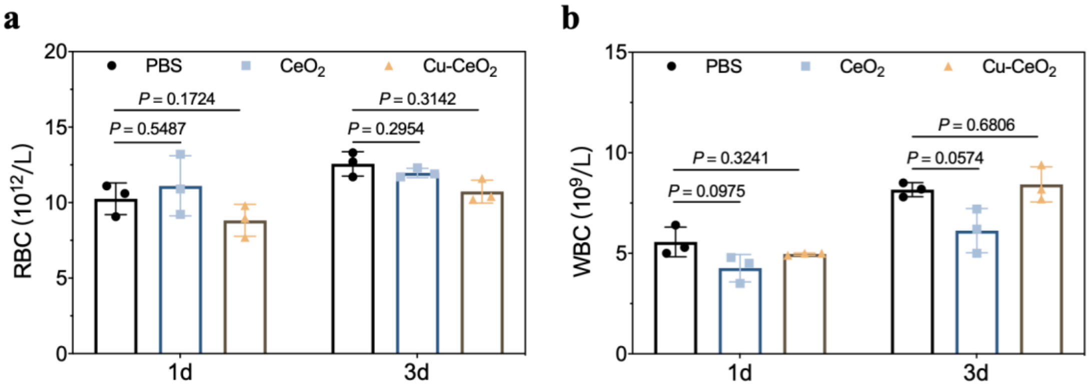

Supplementary Fig. 36. Blood routine results of balb/c mice after intravenous injection with PBS, CeO_2 and Cu-CeO_2. (a) Red blood cells. (b) White blood cells. Data are presented as mean values +/- standard deviation, n = 3 biologically independent replicates. Significance was calculated by two-sided Student's t-test. Source data are provided as a Source Data file.

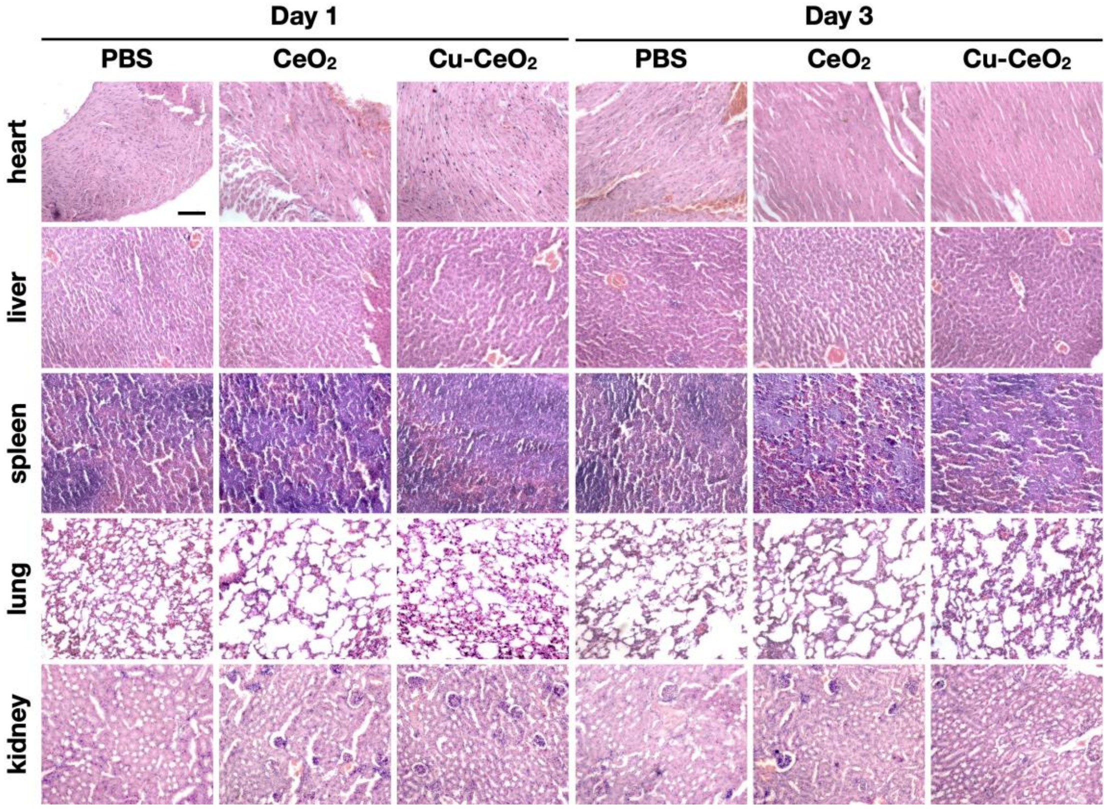

Supplementary Fig. 37. HE staining of the main organs after intravenous injection with PBS, CeO_2 and Cu-CeO_2. Scale bar: 500 µm. A representative image of three replicates from each group is shown.

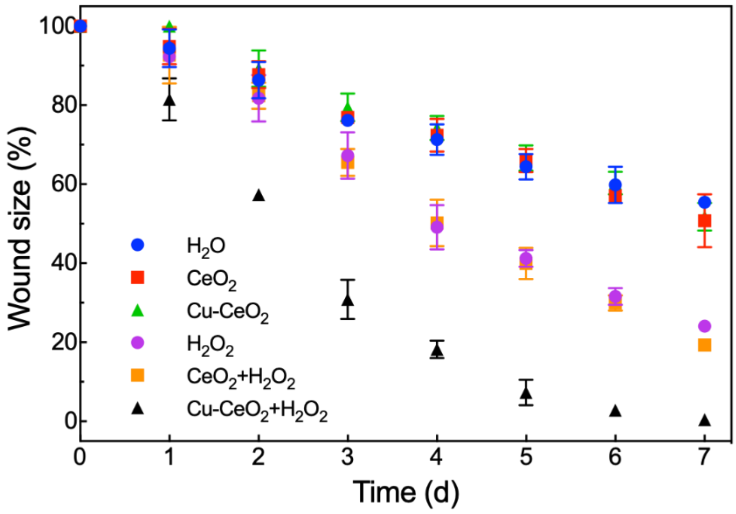

Supplementary Fig. 38. Time-dependent relative wound size after different treatments. Data are presented as mean values +/- standard deviation, n = 3 biologically independent replicates. Source data are provided as a Source Data file.

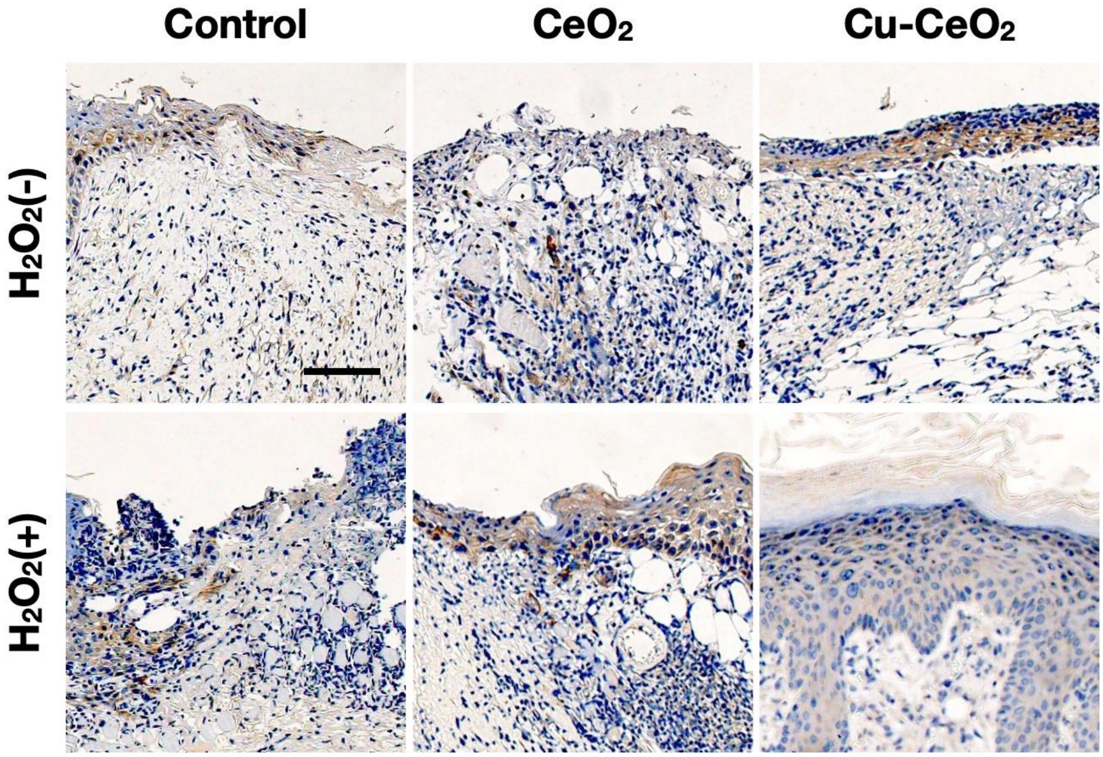

Supplementary Fig. 39. Immunohistochemsistry staining for TNF-α expression in the wound tissues of different groups. Scale bar: 500 µm. A representative image of three replicates from each group is shown.

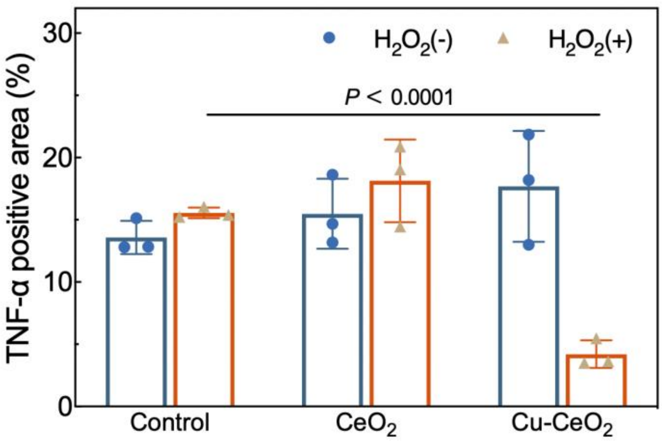

Supplementary Fig. 40. Quantitive analysis of TNF-α expression of different groups. Data are presented as mean values +/- standard deviation, n = 3 biologically independent replicates. Significance was calculated by two-sided Student's t-test. Source data are provided as a Source Data file.

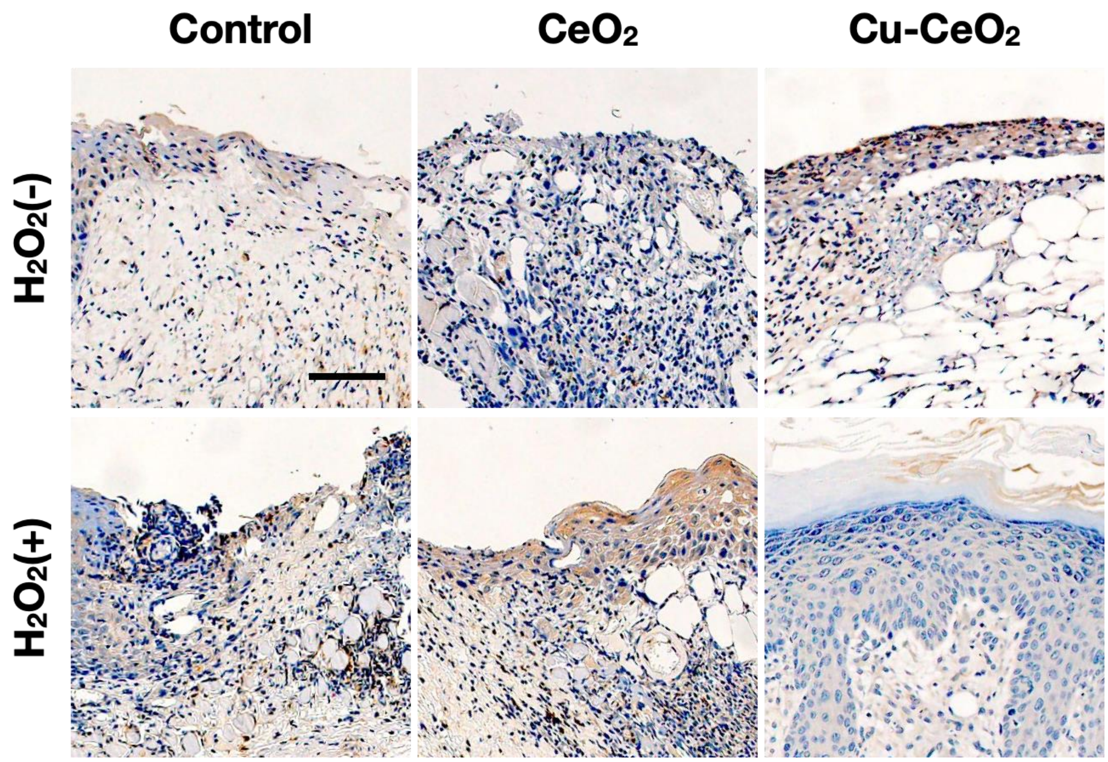

Supplementary Fig. 41. Immunohistochemsistry staining for IL-1β expression in the wound tissues of different groups. Scale bar: 500 µm. A representative image of three replicates from each group is shown.

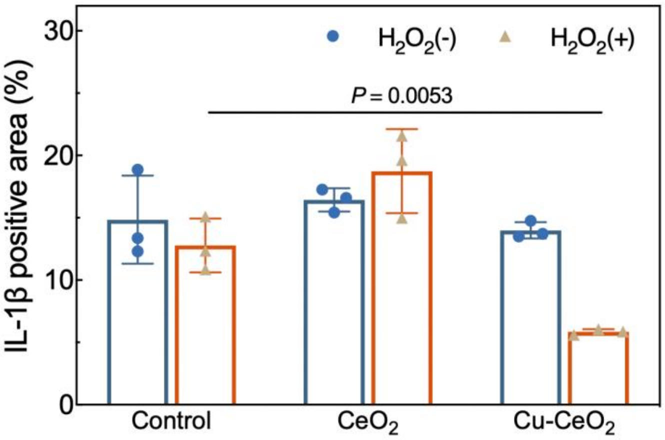

Supplementary Fig. 42. Quantitive analysis of IL-1β expression of different groups. Data are presented as mean values +/- standard deviation, n = 3 biologically independent replicates. Significance was calculated by two-sided Student's t-test. Source data are provided as a Source Data file.
