# Supplementary material for: Tuning oxidant and antioxidant activities of ceria by anchoring copper single-site for antibacterial application
Source: Nat Commun. 2024 Feb 3;15:1010. doi: 10.1038/s41467-024-45255-6 (PMC10837451; doi:10.1038/s41467-024-45255-6)
Supplement: Supplementary file 2 — Reporting Summary [file 41467_2024_45255_MOESM2_ESM.pdf]

nature portfolio

Corresponding author(s): Ting Tan, Dingsheng Wang, Yuguang Wang

Last updated by author(s): Jan 15, 2024

Reporting Summary

Nature Portfolio wishes to improve the reproducibility of the work that we publish. This form provides structure for consistency and transparency in reporting. For further information on Nature Portfolio policies, see our Editorial Policies and the Editorial Policy Checklist.

Statistics

For all statistical analyses, confirm that the following items are present in the figure legend, table legend, main text, or Methods section.

n/a	Confirmed	
☐	☒	The exact sample size (*n*) for each experimental group/condition, given as a discrete number and unit of measurement
☐	☒	A statement on whether measurements were taken from distinct samples or whether the same sample was measured repeatedly
☐	☒	The statistical test(s) used AND whether they are one- or two-sided<br>*Only common tests should be described solely by name; describe more complex techniques in the Methods section.*
☒	☐	A description of all covariates tested
☐	☒	A description of any assumptions or corrections, such as tests of normality and adjustment for multiple comparisons
☐	☒	A full description of the statistical parameters including central tendency (e.g. means) or other basic estimates (e.g. regression coefficient) AND variation (e.g. standard deviation) or associated estimates of uncertainty (e.g. confidence intervals)
☐	☒	For null hypothesis testing, the test statistic (e.g. *F*, *t*, *r*) with confidence intervals, effect sizes, degrees of freedom and *P* value noted<br>*Give P values as exact values whenever suitable.*
☒	☐	For Bayesian analysis, information on the choice of priors and Markov chain Monte Carlo settings
☒	☐	For hierarchical and complex designs, identification of the appropriate level for tests and full reporting of outcomes
☒	☐	Estimates of effect sizes (e.g. Cohen's *d*, Pearson's *r*), indicating how they were calculated

Our web collection on statistics for biologists contains articles on many of the points above.

Software and code

Policy information about availability of computer code

Data collection	The data were collected on Hitachi-7650 (TEM), JEOL JEM-2100F field-emission electron microscope (HRTEM and EDS), JEM-ARM200F (HAADF-STEM), Rigaku RU-200b X-ray diffractometer (XRD), Renishaw inVia micro-Raman spectrometer (Raman), ULVAC PHI Quantera microscope (XPS), Bruker Vertex 70 FTIR spectrometer (FT-IR), Thermo Scientific XSeries 2 (ICP-MS), soft X-ray magnetic circular dichroism end station (National Synchrotron Radiation Laboratory , Hefei, XANES) and beamline BL11B station (Shanghai Synchrotron Radiation Facility, XAFS). Ocular 2.0.1.496 software was used to collect the fluorescent images. CMax Pro 5.1.3 software was used to collect the absorbance data.
Data analysis	GraphPad Prism 7.0 software and IBM SPSS Statistics 24.0 software were used for statistical analyses. Image-Pro Plus 6.0 software was used for quantitative analysis of immnohistochemistry assays. The theoretical X-ray diffraction pattern was simulated using MAUD version 2.8 (Materials Analysis Using Diffraction). The acquired XAFS data was processed using the ATHENA module in the Demeter 0.9.25 software package. Microsoft Excel 2016, Origin 2020, XPSPEAK41 and DigitalMicrograph were used to analyzed the data.

For manuscripts utilizing custom algorithms or software that are central to the research but not yet described in published literature, software must be made available to editors and reviewers. We strongly encourage code deposition in a community repository (e.g. GitHub). See the Nature Portfolio guidelines for submitting code & software for further information.

Data

Policy information about availability of data

All manuscripts must include a data availability statement. This statement should provide the following information, where applicable:

- Accession codes, unique identifiers, or web links for publicly available datasets
- A description of any restrictions on data availability
- For clinical datasets or third party data, please ensure that the statement adheres to our policy

All data are available within the Article and Supplementary Files (Supplementary Information, Supplementary Data 1), or available from the corresponding authors upon request. Source data are provided with this paper.

Research involving human participants, their data, or biological material

Policy information about studies with human participants or human data. See also policy information about sex, gender (identity/presentation), and sexual orientation and race, ethnicity and racism.

Reporting on sex and gender	NA
Reporting on race, ethnicity, or other socially relevant groupings	NA
Population characteristics	NA
Recruitment	NA
Ethics oversight	NA

Note that full information on the approval of the study protocol must also be provided in the manuscript.

Field-specific reporting

Please select the one below that is the best fit for your research. If you are not sure, read the appropriate sections before making your selection.

☒ Life sciences ☐ Behavioural & social sciences ☐ Ecological, evolutionary & environmental sciences

For a reference copy of the document with all sections, see nature.com/documents/nr-reporting-summary-flat.pdf

Life sciences study design

All studies must disclose on these points even when the disclosure is negative.

Sample size	For animal experiments, the minimum number of animals of different groups were n≥3 to achieve statistical significance. For cell-based quantitative experiments, results of three independent replicates were used. Sample size of each experiment is indicated in Figure legends.
Data exclusions	No data exclusion.
Replication	Presented experiments were repeated for three times independently. All attempts at replication were successful.
Randomization	For cell-based and animal experiments, the samples were randomly divided for different treatments.
Blinding	The researcher performing the animal experiment was blind for the animal groups. The groups were code labeled other than labeled by treatments, which were reveled after results analysis. Another researcher was blinded during data collection and analysis in the animal experiments.

Reporting for specific materials, systems and methods

We require information from authors about some types of materials, experimental systems and methods used in many studies. Here, indicate whether each material, system or method listed is relevant to your study. If you are not sure if a list item applies to your research, read the appropriate section before selecting a response.

Materials & experimental systems

n/a	Involved in the study	
	x	Antibodies
x		Eukaryotic cell lines
x		Palaeontology and archaeology
	x	Animals and other organisms
x		Clinical data
x		Dual use research of concern
x		Plants

Methods

n/a	Involved in the study	
x		ChIP-seq
x		Flow cytometry
x		MRI-based neuroimaging

Antibodies

Antibodies used	Anti-TNFa alpha antibody (Abcam, ab307164)<br>IL-1 beta Polyclonal Antibody (Invitrogen, P420B)
Validation	All primary antibodies were confirmed or the species and application through the validation statement on the manufacturer's website and their use in the literature.<br>Anti-TNF alpha antibody (Abcam, ab307164) https://www.abcam.com/products/primary-antibodies/tnf-alpha-antibody-rm1005-ab307164.html<br>IL-1 beta Polyclonal Antibody (Invitrogen, P420B) https://www.thermofisher.cn/cn/zh/antibody/product/IL-1-beta-Antibody-Polyclonal/P420B

Animals and other research organisms

Policy information about studies involving animals; ARRIVE guidelines recommended for reporting animal research, and Sex and Gender in Research

Laboratory animals	6-week-old BALB/c mice, all SPF grade, fed in a barrier environment. Mice were kept in constant temperature (22 ℃), constant humidity (55%) and cyclic lighting (12 h light/12 h dark).
Wild animals	No wild animals were used in the study.
Reporting on sex	This study only included female mice, and we did not pay attention to the effect of gender on wound healing. Since male mice are more prone to bite wounds and dressings than female mice when fed in the same cage, we chose more docile female mice.
Field-collected samples	No field-collected samples involved.
Ethics oversight	All animal experiments were carried out under the approval of the biomedical ethics committee of Peking University.

Note that full information on the approval of the study protocol must also be provided in the manuscript.

Plants

Seed stocks	NA
Novel plant genotypes	NA
Authentication	NA
