# Supplementary material for: Tuning oxidant and antioxidant activities of ceria by anchoring copper single-site for antibacterial application
Source: Nat Commun. 2024 Feb 3;15:1010. doi: 10.1038/s41467-024-45255-6 (PMC10837451; doi:10.1038/s41467-024-45255-6)
Supplement: Supplementary file 4 — Description of Additional Supplementary Files [file 41467_2024_45255_MOESM4_ESM.pdf]

Description of Additional Supplementary files

File name: Supplementary Data 1

Description: The compressed file ***Supplementary Data 1.zip*** contains all the DFT optimized structures of the reaction intermediates in ***.vasp*** format, which are also illustrated in **Fig. S21-28**.
